# Mucosal glycan degradation of the host by the gut microbiota

**DOI:** 10.1093/glycob/cwaa097

**Published:** 2020-10-09

**Authors:** Andrew Bell, Nathalie Juge

**Affiliations:** The Gut Microbes and Health Institute Strategic Programme, Quadram Institute Bioscience, Rosalind Franklin Road Norwich Research Park, Norwich NR4 7UQ, UK; The Gut Microbes and Health Institute Strategic Programme, Quadram Institute Bioscience, Rosalind Franklin Road Norwich Research Park, Norwich NR4 7UQ, UK

**Keywords:** glycosylation, gut microbiota, mucin, mucus

## Abstract

The gut microbiota plays a major role in human health and an alteration in gut microbiota structure and function has been implicated in several diseases. In the colon, mucus covering the epithelium is critical to maintain a homeostatic relationship with the gut microbiota by harboring a microbial community at safe distance from the epithelium surface. The mucin glycans composing the mucus layer provide binding sites and a sustainable source of nutrients to the bacteria inhabiting the mucus niche. Access to these glycan chains requires a complement of glycoside hydrolases (GHs) produced by bacteria across the phyla constituting the human gut microbiota. Due to the increased recognition of the role of mucus-associated microbes in human health, how commensal bacteria breakdown and utilize host mucin glycans has become of increased interest and is reviewed here. This short review provides an overview of the strategies evolved by gut commensal bacteria to access this rich source of the nutrient with a focus on the GHs involved in mucin degradation.

## Introduction

The gastrointestinal (GI) tract is home to a diverse range of microbial species collectively referred to as the gut microbiota which have a profound impact on host health. It is well established that the gut microbiota aids digestion of complex dietary polysaccharides which reach the colon undigested, enabled by the vast array of glycolytic enzymes encoded by gut symbionts (Zimmermann *et al.* 2019, El Kaoutari *et al.* 2013). In addition to dietary polysaccharides, gut microbes can utilize host glycans as a nutrient source. The ability to metabolize glycans such as human milk oligosaccharides, glycosaminoglycans and glycan moieties of glycoproteins and glycolipids found at mucosal surfaces grants bacteria a competitive advantage. This is particularly relevant to the microbial community that resides within the mucus layer of the large intestine.

The mucus layer is viewed as a defence mechanism, protecting the epithelial layer from microbes and other luminal compounds, but in the colon, mucus also plays a major biological function by harboring a distinct microbial community called the mucus-associated microbiota. This is enabled by the bilayer organization of the colonic mucus which is divided into a stratified inner layer virtually impenetrable to bacteria and a loose outer layer providing a niche to microbes adapted to this environment (Johansson *et al.* 2008). This microbial community is tolerated due to the mutually beneficial relationship established with the host as a result of long-term coevolution (Neish 2009). Benefit to the host includes effective mucin turnover and stimulation of mucus production through Toll-like receptor-mediated interactions with sentinel goblet cells (Birchenough *et al.* 2016). Continuous mucus production is essential to maintain gut barrier function and is strengthened by the production of antimicrobial compounds against pathogenic bacteria (McGuckin *et al.* 2011). Other benefits of the mucus-associated microbiota include colonization resistance whereby pathogenic niches are already occupied by commensal species (Sorbara and Pamer 2019), and the production of metabolites directly implicated in the communication of microbes with the host. The mucus-associated gut microbiota can also significantly affect the development of the host immune system as extensively reviewed (Pickard *et al.* 2017).

## Mucin glycosylation and associated bacteria in the gut

Mucin glycans make up ~80% of the molecular mass of mucins, the main structural component of mucus. Mucin-type *O*-glycosylation is initiated by a large family of polypeptide GalNAc transferases (ppGalNAc Ts) that add α-GalNAc to the Ser and Thr residues of peptides. Mucin glycosylation is characterized by a high degree of structural diversity which is based on three elements. The first is the type of core structure. There are eight mucin core structures in humans with structures 1–4 most commonly found in intestinal mucins (Tailford *et al.* 2015a; Thomsson *et al.* 2012; Brockhausen *et al.* 2009). The second stage of glycan diversity is determined by the action of a range of glycosyltransferases that elongate the mucin core through the addition of galactose, *N*-acetylgalactosamine (GalNAc) and/or *N-*acetylglucosamine (GlcNAc) residues leading to linear or branched chains of up to 20 residues (Gunning *et al.* 2013). The third element of diversity is conferred by the peripheral epitopes that are often fucosylated, sialylated or sulphated (Tailford *et al.* 2015a).

At the ecological level, the diversity of mucin glycans along the GI tract contributes to shape the structure and function of the gut microbiota. While the luminal microbiota may respond primarily to diet, the mucus-associated microbiota is influenced more directly by host-related factors. Importantly, the ability to utilize host mucin glycans as a carbon source gives bacteria a sustainable and consistent nutrient supply and a competitive advantage to colonize the mucus layer (Marcobal *et al.* 2013). As reviewed in Tailford et al. (2015a), it is now established that mucin glycan degradation is widespread across the major phyla represented in the human gut microbiota. *Akkermansia muciniphila* is a mucin glycan degradation specialist and, therefore, considered as a keystone member of the mucus-associated microbiota (Shin *et al.* 2019) while Bacteroidetes are viewed as general glycan degraders able to switch from dietary to host glycan metabolism due to their extensive array of carbohydrate-active enzymes (Ndeh and Gilbert 2018). Actinobacteria, which are largely represented by *Bifidobacteria* in the human gut microbiota, are typically adapted to carbohydrates with a low degree of polymerization and mucin glycan metabolism strategy is similar to the Firmicutes (Ndeh and Gilbert 2018). Consistent with this, the presence of mucins in *in vitro* fermentation models leads to an increased proportion of *Bacteroidetes, Akkermansia* and *Lachnospiraceae* species that are known mucin glycan degraders, whilst levels of *Lactobacillus* and *Bifidobacterium* decrease (Tran *et al.* 2016). *In vivo*, both chronic and intermittent fiber deficiency promotes enrichment of mucin glycan degrading bacteria in mouse models, leading to a significant increase in *A. muciniphila* and *Bacteroides caccae* species accompanied by a decrease of the fiber-degrading species (Desai *et al.* 2016).

## Mucin glycan degradation strategies by gut commensal bacteria

**Fig. 1:**
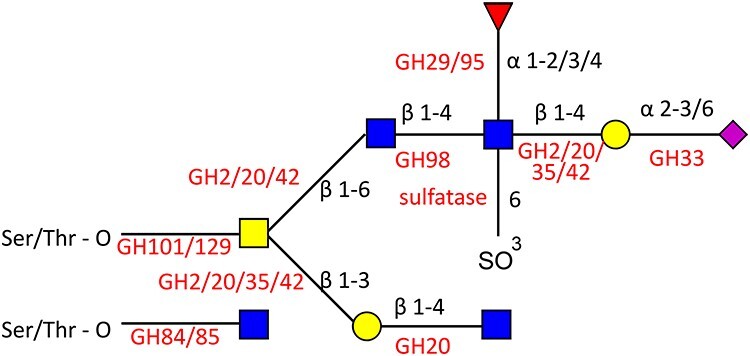
Specificity of bacterial GHs and sulfatases. A hypothetical mucin glycan is depicted with monosaccharide symbols following the symbol nomenclature for glycans (Varki *et al.*, 2015). Linkages are shown with black text and GHs are shown in red text.

Microbes most adept at mucin glycan degradation often encode sulfatases, deacetylases, sialidases and fucosidases to remove terminal structures and grant greater accessibility to the extended core structures (Etienne-Mesmin *et al.* 2019; Ndeh and Gilbert 2018). The monosaccharides freed by the action of these enzymes may be utilized by the bacteria themselves or released in the environment for scavenging bacteria (Marcobal *et al.* 2013). Furthermore, *in silico* analysis revealed that up to 86% of the human gut microbiota encode genes for cleavage of mucin glycans, with 89% encoding genes for the metabolism of the monosaccharides released (Ravcheev and Thiele 2017). The current model for mucin glycan degradation by the gut microbiota involves the sequential action of a number of glycoside hydrolases (GHs) (www.cazy.org; Figure 1) (Lombard *et al.* 2014).

Sulfate residues terminate mucin glycans and have been proposed to prevent GHs from removing terminal sugars, thus preventing the breakdown of mucin glycans (Etienne-Mesmin *et al.* 2019). In addition, the release of sulfate residues has been proposed to increase the levels of sulfate-reducing bacteria in the gut, leading to the production of H_2_S, which can disrupt the mucus network and lead to epithelial damage (Praharaj *et al.* 2018; Ijssennagger *et al.* 2016). Mucin-desulfating enzymes have been characterized primarily from the Bacteroides genus, with examples from *B. fragilis* and *B. thetaiotaomicron* (Praharaj *et al.* 2018; Cartmell *et al.*, 2017). Recent work identified a *B. fragilis* sulfatase that was shown to be essential for growth on mucus *in vitro* and robust mucosal colonization *in vivo* (Donaldson *et al.*, 2020).

Exo-acting GHs are then involved in the trimming of terminal sugars from the *O*-glycan mucin chains, starting with the removal of fucose and sialic acid residues capping the GI mucin chains.

Fucose release involves fucosidases belonging to GH29 and GH95 families (www.cazy.org). GH95 enzymes functionally characterized so far show strict substrate specificity to the terminal Fucα1–2Gal linkage and hydrolyse the linkage *via* an inverting mechanism whereas GH29 enzymes show relatively relaxed substrate specificities with hydrolysis proceeding *via* a retaining mechanism (www.cazy.org). Fucosidases are found among numerous members of the gut microbiota, and often multiple fucosidases are found within a single genome, for example *Bifidobacterium bifidum* (Ashida *et al.*, 2009)*, Bifidobacterium longum* (Garrido *et al.*, 2016, Bunesova *et al.* 2016), *Ruminococcus gnavus* (Crost *et al.* 2013) or *A. muciniphila* (Ottman *et al.* 2017). In these species, transcriptomics studies demonstrated that fucosidases were upregulated during growth on mucins, supporting their role in mucin glycan breakdown and utilization (Shin *et al.* 2019; Crost *et al.* 2016). Fucose metabolism has also been demonstrated for *B. thetaiotaomicron* and can trigger host fucosylation which *B. thetaiotaomicron* then uses as a nutrient source (Pickard and Chervonsky 2015). Fucose and mucin cross-feeding initiated by *B. bifidum* enables growth of *Eubacterium hallii*, an early occurring commensal species that produces butyrate and propionate from fermentation metabolites but that cannot degrade complex oligo- and polysaccharides (Bunesova *et al.* 2018; Schwab *et al.* 2017). However, not all fucosidases are extracellular, for example, 3 intracellular fucosidases with varying substrate specificities toward disaccharides have been characterized from lactobacilli (AlfA, AlfB and AlfC) (Rodríguez-Díaz *et al.* 2011), suggesting that Lactobacilli may import fucosyl-oligosaccharides.

Sialic residues are another highly sought-after source of nutrient terminating mucin glycan chains. The sialic acids comprise a family of 9-carbon sugar acids found predominantly on cell surface glycans of humans and other animals, the most common form of sialic acid in humans is *N*-acetylneuraminic acid (Neu5Ac). To access this carbon and nitrogen source, intestinal bacteria (both gut symbionts and pathogens) express GH33 sialidases (also known as neuraminidases), which cleave terminal sialic acid residues. Several sialidases have been functionally and structurally characterized from gut bacteria including species of Clostridia (Navarro *et al.* 2018) and Bacteroidetes, such as *Bacteroides fragilis or Bacteroides thetaiotamicron* (Juge *et al.* 2016), as well as specific strains of Bifidobacterium (Nishiyama *et al.* 2018), *R. gnavus* (Tailford *et al.* 2015b) and *A. muciniphila* (Huang *et al.* 2015a). *B. fragilis* sialidase preferentially cleaves the sialyl α2,8 linkage compared to sialyl α2,3 and α2,6 linkages that are more commonly targeted (Tanaka *et al.* 1994). These sialidases are usually extracellular so the sialic acid is released into the environment, where it can be imported by the bacteria or scavenged by other microbes including strains from the same species. The action of two sialidases from *B. bifidium* was shown to support the growth of *Bifidobacterium breve* through sialic acid crossing-feeding (Nishiyama *et al.* 2018). Interestingly, some bacteria, such as *B. thetaiotaomicron* ATCC 29148 or *A. muciniphila* DSM 22959 (Tailford *et al.* 2015b; Huang *et al.*, 2015a) encode sialidases allowing access to the underlying sugars of the glycan chains but do not encode genes required for sialic acid utilization (Brigham *et al.* 2009). In certain conditions such as post-antibiotic treatment, the levels of free sialic acid can promote the expansion of pathogens such as *Clostridium difficile*, *Salmonella* and *Escherichia coli* that do not produce sialidases (Ng *et al.* 2013). Another study showed that the expansion of certain *Bacteroides* species in a mouse model of colitis led to increased levels of sialidases and subsequent outgrowth of *E. coli*, which was dependent on the ability to catabolize sialic acid (Huang *et al.* 2015b). It is believed that *O*-acetyl ester modifications of sialic acids present at high levels in the mammalian colon can help protect from the action of bacterial sialidases (Robinson *et al.* 2017). In turn, some gut bacteria, produce sialylate-*O*-acetylesterases to remove them. *In vitro* foraging studies demonstrated that sialidase-dependent *E. coli* growth on mucin is enabled by *Bacteroides* EstA, a sialate *O*-acetylesterase acting on glycosidically linked sialylate-*O*-acetylesterase substrates (Robinson *et al.* 2017). It was, therefore, proposed that EstA specifically unlocks the nutritive potential of 9-O-acetylated sialic acids in mucus for mucin glycan foraging bacteria. Interestingly, *R. gnavus* encodes an intramolecular-*trans*-sialidase which releases 2,7-anhydro-Neu5Ac instead of Neu5Ac released by hydrolytic sialidases (Tailford *et al.* 2015b). This is proposed to be part of a selfish mechanism employed by *R. gnavus* to hold on to sialic acid by releasing it in a form only it can preferentially access and utilize (Bell *et al.* 2020). The full metabolic pathway for the utilization of 2,7-anhydro-Neu5Ac was recently unraveled and it was shown that the pathway was intrinsically linked to mucosal colonization by *R. gnavus* in mouse models (Bell *et al.* 2020).

Following removal of terminal sugars, GHs including galactosidases (GH2, GH20, GH35, GH42, GH98), *N*-acetylglucosaminidases (GH84, GH85, G89) and *N*-acetylgalactosaminidases (GH101, GH129) can degrade the extended core structures, releasing free monosaccharides that can support growth of bacteria (Figure 1) (Tailford *et al.* 2015a; Marcobal *et al.* 2013). Recently three mucin-acting extracellular β-galactosidases (GH2, GH35) from *A. muciniphila* ATCC BAA-835 were characterized with varied specificity toward glycosidic linkages. Amuc_0824 (GH2) was primarily active against Galβ1-3GalNAc whereas Amuc_0771 (GH35) showed the greatest activity against lacto*-N*-biose and galacto*-N*-biose (Kosciow and Deppenmeier 2020). In contrast, Amuc_1666 (GH2) showed activity against β1–4 linkages. These linkages are highly abundant in mucin glycans showing the importance of these enzymes in the mucin degradation strategy of *A. muciniphila.* Such diversity in substrate specificity is also seen across the *Bifidobacteria* from the human infant gut. Bioinformatics analyses revealed that β-galactosidase activity is spread across *Bifidobacteria* with certain clusters of β-galactosidase being strain-specific while others appeared to be shared across *Bifidobacteria.* Characterization of representative β-galactosidases of each cluster confirmed unique patterns of substrate specificity, with broad substrate specificity enzymes found across all subspecies (Ambrogi *et al.* 2019). In addition to exo-acting β-galactosidases endo-acting β-galactosidases (GH98) have also been described, for example, *eabC* from *Clostridium perfringens* is shown to able to cleave off blood group antigens (see recent review, Low *et al.* 2020).

Glucosaminidases that act on GlcNAc residues are found in multiple GH families. Exo-β-*N*-acetylglucosaminidases (GH84) have been identified across members of the gut microbiota (www.cazy.org), with some species encoding multiple enzymes. The substrate specificity of GH84 enzymes can include β1–2, 1–3, 1–4 and 1–6 linkages (Pluvinage *et al.* 2019). Endo- β-*N*-acetylglucosaminidases (GH85) that cleave the chitobiose core (GlcNAc-β-1,4-GlcNac) are also widespread in bacteria, and show a strict preference for GalNAc, and both core 1 and core 3 can be cleaved by these enzymes (Koutsioulis *et al.* 2008). Recent crystallographic evidence showed that GlcNAc was also the natural ligand for members of the GH20 family in *A. muciniphila* (Chen *et al.* 2019), a family containing exo-acting β -*N-*acetylglucosaminidases, β -*N*-acetylgalactosamindase, β −6-SO_3_-*N*-acetylglucosaminidases, and exo-acting lacto-*N*-biosidases (www.cazy.org).

The GH101 family regroups enzymes responsible for cleaving the core-1 *O*-linked glycans (Gal-β-1,3-GalNAc-α-R) with some of the family members shown to have some degree of activity against core 2 and core 3 structures (Koutsioulis *et al.* 2008). The α-*N*-acetylgalactosaminidases belonging to family GH129 show sequence similarity to GH101 members; however, they have a distinct substrate specificity, favoring the GalNAc-α1-Ser Tn antigen structure found in mucin glycoproteins. They are abundant among *Bifidobacteria* species and act intracellularly suggesting transport of Tn antigen containing oligosaccharides in the bacteria (Kiyohara *et al.* 2012). The first crystal structure from the GH129 family showed structural similarities with GH101 but differences in substrate recognition account for the altered substrate specificity (Sato *et al.* 2017).

In Bacteroides, oligosaccharides are imported in the periplasm where they are further degraded, and the enzymes to do this are physically linked into loci termed polysaccharide utilization loci (PULs) (Brown and Koropatkin 2020, Lapébie *et al.* 2019). In addition to the exo-acting GHs reported above and consistent with the glycan degradation strategy in these species, recent studies reported endo-acting enzymes that target the polyLacNAc structures within oligosaccharide side chains of mucins. These *O*-glycanases are found in several Bacteroides spp. as well as *A. muciniphila* and are a part of the GH16 family (Crouch *et al.* 2019). In addition, a high throughput screening approach led to the identification of novel GH31 and GH109 enzymes with α-GalNAcase activity (Rahfeld *et al.* 2019). These enzymes were found to have distinct specificities toward mucin-type *O*-glycans and blood type A-antigens. The α-GalNAcase GH31 enzymes act solely upon the GalNAc present in core structures of mucin-type *O*-glycans with no activity toward blood type A-antigens. The putative PULs in which the described α-GalNAcase GH31 enzymes are located showed no similarity to known mucin-degrading PULs (Rahfeld *et al.* 2019). It has been proposed that GH31 family enzymes may therefore play a major role in the capacity of Bacteroides spp. to efficiently degrade mucosal glycans despite their lack of GH101 or GH129 family enzymes (described above) (Rahfeld *et al.* 2019).

## Perspectives

With the field of gut microbiota expanding beyond association studies and the increasing acknowledgment of the role of mucus-associated bacteria in human health, it is critical to continue our effort to gain mechanistic insights into the mechanisms underpinning microbial degradation of host mucin glycans. A full integration of glycomics in the field of microbiome research is warranted to further our understanding of the function and adaptation of microbial communities within the distinct nutritional niches in the gut. Combined with relevant *in vivo* humanized mouse models and advanced biopsy-based *in vitro* organ cultures, this biochemical knowledge will help to provide tangible molecular leads for developing therapeutic strategies to modulate the gut microbiota at the mucosa surface and strengthen gut barrier function in humans. Together, these targeted and omics approaches will potentiate the translation of microbiome research for biomarker development and precision medicine.
