# Corrigendum to: Mucosal glycan degradation of the host by the gut microbiota

**DOI:** 10.1093/glycob/cwac008

**Published:** 2023-10-01

In the originally published version of this manuscript, in the section “Mucin glycan degradation strategies by gut commensal bacteria,” the last sentence of the section, on page 694, has been updated.

